# Cellular Senescence in Kidney Fibrosis: Pathologic Significance and Therapeutic Strategies

**DOI:** 10.3389/fphar.2020.601325

**Published:** 2020-12-11

**Authors:** Jie Xu, Lili Zhou, Youhua Liu

**Affiliations:** ^1^ State Key Laboratory of Organ Failure Research, National Clinical Research Center of Kidney Disease, Nanfang Hospital, Southern Medical University, Guangzhou, China; ^2^ Department of Pathology, University of Pittsburgh School of Medicine, Pittsburgh, PA, United States

**Keywords:** senescence, premature aging, chronic kidney disease, kidney fibrosis, senescence-associated secretory phenotype, senotherapy

## Abstract

Age-related disorders such as chronic kidney disease (CKD) are increasingly prevalent globally and pose unprecedented challenges. In many aspects, CKD can be viewed as a state of accelerated and premature aging. Aging kidney and CKD share many common characteristic features with increased cellular senescence, a conserved program characterized by an irreversible cell cycle arrest with altered transcriptome and secretome. While developmental senescence and acute senescence may positively contribute to the fine-tuning of embryogenesis and injury repair, chronic senescence, when unresolved promptly, plays a crucial role in kidney fibrogenesis and CKD progression. Senescent cells elicit their fibrogenic actions primarily by secreting an assortment of inflammatory and profibrotic factors known as the senescence-associated secretory phenotype (SASP). Increasing evidence indicates that senescent cells could be a promising new target for therapeutic intervention known as senotherapy, which includes depleting senescent cells, modulating SASP and restoration of senescence inhibitors. In this review, we discuss current understanding of the role and mechanism of cellular senescence in kidney fibrosis. We also highlight potential options of targeting senescent cells for the treatment of CKD.

## Introduction

Chronic kidney disease (CKD) afflicts more than 10% population and is becoming a major public health problem worldwide (Denic et al., 2016; Yang et al., 2020). The prevalence of CKD in the elderly reaches to 14.3–41.3% in some countries (Susnik et al., 2017). CKD is also an independent risk factor for cardiovascular complications and all-cause mortality. The costs for managing CKD patients are staggering, which imposes tremendous burdens to the afflicted individuals, families and societies, respectively (Wang et al., 2016). At present, there is no curative treatment for CKD. Current therapies include lifestyle and dietary modifications, blood pressure management by blocking the renin angiotensin aldosterone system (RAAS), and glycemic control (Breyer and Susztak, 2016; Kalantar-Zadeh and Fouque, 2017; Ruiz-Ortega et al., 2020). These remedies at best only slow, but not completely halt, CKD progression, and they are often ineffective and possess adverse side effects. In this context, it is paramount and urgent to elucidate the patho-mechanisms of CKD and to develop new therapeutic strategies.

Increasing evidence indicates that there is a striking similarity between the manifestations of progressive CKD and aging kidney (Docherty et al., 2020; Goligorsky, 2020; Zhou et al., 2020). As such, CKD is often viewed as a form of premature and accelerated aging. Aging and CKD also share many common triggers and underlying mechanisms, such as cellular senescence, oxidative stress, inflammation, mitochondrial dysfunction, RAAS activation and hyperactive Wnt/β-catenin (Sturmlechner et al., 2017; Xiong and Zhou, 2019). In various animal models and human kidney biopsies, accumulation of senescent cells in different renal compartments is increasingly recognized as a common pathway leading to premature aging and CKD (Docherty et al., 2019; Docherty et al., 2020).

Cellular senescence is characterized by an irreversible and permanent cell cycle arrest coupled with altered transcriptome and secretome. Unlike apoptosis, senescence is a state of which cells are still alive and metabolically active. Senescence is a complex and evolutionarily conserved program that plays both beneficial and detrimental roles under different circumstances. Generally speaking, acute and transient cellular senescence makes positive contributions to various biological processes such as embryogenesis, tissue remodeling, injury repair and tumor suppression (Storer et al., 2013; Gonzalez et al., 2016). However, chronic and uncontrolled senescence after injury inevitably results in premature aging, tissue fibrosis and organ dysfunction (Melk et al., 2004).

In this review, we summarize the evidence linking cellular senescence to the pathogenesis of CKD and discuss current understanding of the mechanism and regulators controlling senescence. We also highlight potential options for targeting senescent cells in developing therapeutics for CKD patients.

## CKD AS A STATE OF PREMATURE AGING

CKD is defined as gradual loss of kidney function for at least 3 months duration, regardless of etiologies (Webster et al., 2017). In developed countries, diabetic nephropathy (DN) is the leading cause of CKD, while glomerulonephritis is much more common in most developing countries (Barsoum, 2006; Yang et al., 2020). Histopathologically, CKD is characterized by activation of α-smooth muscle actin (α-SMA)-positive myofibroblasts, excessive production and deposition of extracellular matrix (ECM) leading to tissue fibrosis, infiltration of inflammatory cells, tubular atrophy and microvascular rarefaction (Liu, 2011; Humphreys, 2018; Yuan et al., 2019).

**FIGURE 1:**
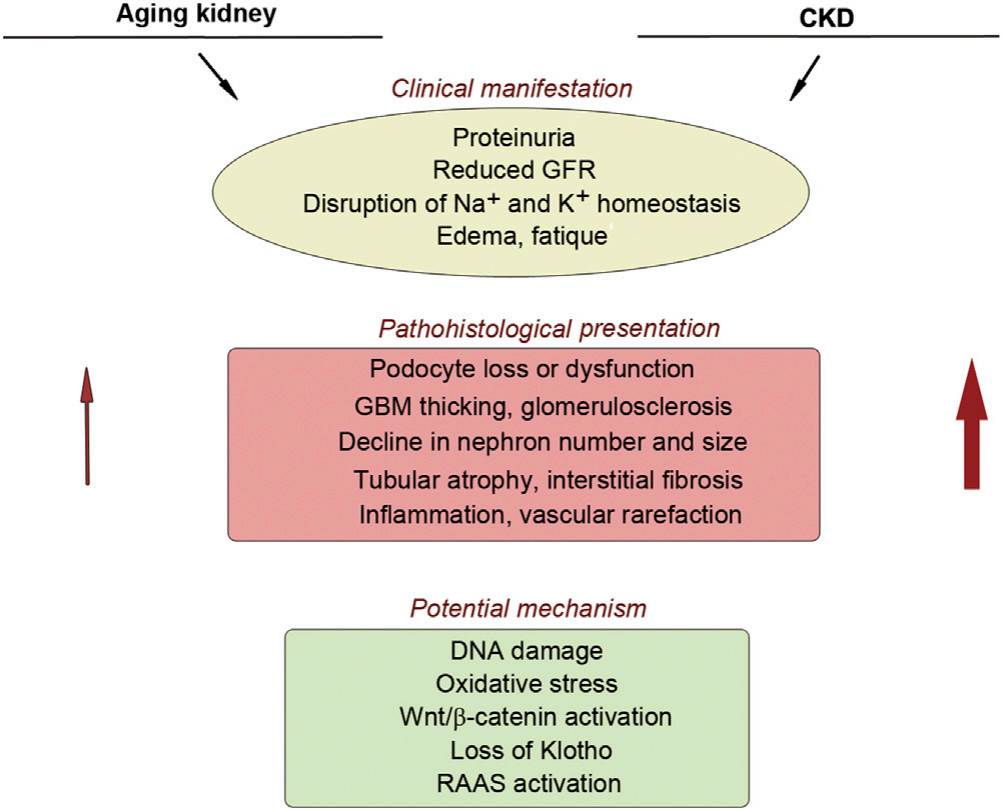
Similarity between aging kidney and chronic kidney disease. There is a striking similarity in the clinical manifestation, pathohistological presentation, and potential mechanism of nephropathy between aging kidney and CKD. Red arrows show that aging kidney usually displays mild pathological lesions, compared to CKD.

Kidney function also declines progressively with aging. In general, estimated glomerular filtration rate (eGFR) decreases, beginning at approximately age 30, at the rate of 0.7–0.9 ml/min/1.73 m^2^ per year in healthy individuals (Glassock and Rule, 2016). As aging progresses, kidney structure and function undergo a series of changes reminiscent of that in CKD, including reduced number and size of nephrons, glomerulosclerosis, tubular atrophy, inflammation, interstitial fibrosis, as well as increased prevalence of vascular rarefaction and arteriosclerosis (Hodgin et al., 2015; Hommos et al., 2017), although these changes are usually mild compared to CKD (Figure 1). Aging kidneys are also vulnerable to injury and often fail to regenerate and recover (Schmitt and Cantley, 2008; Stenvinkel and Larsson, 2013).

Premature aging is referred to a condition in which changes resembling characteristics of aging are manifested at a very early age. Typically, premature aging is accompanied by hypofunction of multiple organs, vulnerable to injury and high risk of diseases. At the cellular level, accumulation of senescent cells and stem cell exhaustion are typical feature of premature aging (Kooman et al., 2014; Spehar et al., 2020). As a spectrum of intersecting signaling plays a crucial role in regulating cell longevity, any stressors causing disruption or dysregulation of these pathways would result in premature aging, such as DNA damage, oxidative stress, telomere shortening, loss of Klotho, oncogene activation (Kubben and Misteli, 2017; Opresko and Shay, 2017; Rowland et al., 2019).

In many ways, CKD could be considered as a state of premature aging (Stenvinkel and Larsson, 2013), as they share common features (Figure 1). Furthermore, CKD complications include frailty, muscle wasting, vascular calcification, osteoporosis and cardiovascular disorders, which are all characteristic features of aging (Stenvinkel and Larsson, 2013; Kooman et al., 2014; Kooman et al., 2017b). Therefore, a better understanding of the pathophysiological mechanisms underlying cellular senescence and premature aging is essential for delineating the cause and mechanism of CKD and designing interventional strategies.

## CELLULAR SENESCENCE: CHARECTERIZATION AND PATHWAY

### Identification of Senescent Cells

Cellular senescence is defined as a permanent cease of cell cycle progression even in the presence of growth factor stimulation. However, it is challenging to identify senescent cells with certainty *in vivo* due to the lack of a sensitive and specific marker. The characteristic features of senescent cells include their resistance to apoptosis and phenotypic changes such as altered morphology with large flattened cell bodies (Knoppert et al., 2019). As summarized in Table 1, there are several characteristic markers associated with cellular senescence and they are frequently used in the field (Gorgoulis et al., 2019; Docherty et al., 2020). Although a single marker may not be specific and sufficient, combination of several of these markers is considered adequate for identifying senescent cells *in vitro* and *in vivo*.

**TABLE 1:** Commonly used markers for identifying senescent cells.

Markers	Nature	Function
**Overexpressed in senescent cells**		
SA-β-gal	Senescence-associated β-galactosidase	Reflects the enhanced lysosomal content of senescent cells (Luo et al., 2018; Gorgoulis et al., 2019; Docherty et al., 2020)
p16^Ink4a^ p21^Cip1^	Cyclin-dependent kinase (CDK) inhibitor CDK inhibitor	Binds to CDK4 and 6 complex and inhibits its activity, resulting in dephosphorylation of pRb and suppression of G1/S phase (Hernandez-Sequra et al., 2018; Gorgoulis et al., 2019) Mainly inhibits CDK2, inducing cell cycle arrest in G1/S phase and cell senescence (Docherty et al., 2020; Zhou et al., 2020)
ARF	CDK inhibitor alternate reading frame	p14^ARF^ in human, p19^ARF^ in mouse, also block cell cycle at G1/S phase (Luo et al., 2018)
γ-H2AX	The phosphorylated form of H2AX, and a selective marker of DNA double-strand breaks	Through DDR to induce cell cycle arrest at G2/M phase by activating checkpoint kinase 1 and 2 (d'Adda di Fagagna, 2008; Hernandez-Sequra et al., 2018)
SAHF	Senescence-associated heterochromatin foci	Manifests altered DNA packing within senescent cells (Aird and Zhang, 2013)
**Downregulated in senescent cells**		
Ki-67	Cell proliferation-related markers	A nuclear protein associates with cell proliferation, decreased in senescent cells
LMNB1	Nuclear lamina protein lamin B1	Loss of lamin B1 promotes cellular senescence via a p53- and Rb-dependent mechanism (Hernandez-Sequra et al., 2018; Lukasova et al., 2018)

One widely used marker for senescent cells is the increased senescence-associated β-galactosidase (SA-β-Gal), which reflects the enhanced lysosomal content of senescent cells. However, it should be noted that SA-β-Gal activity itself may not be necessary for cellular senescence to occur (Hernandez-Sequra et al., 2018) and it is also increased in non-senescent confluent cells with high density in cell cultures, which limits its use as a standalone marker for detecting senescent cells. Furthermore, SA-β-Gal activity cannot be used for paraffin-embedded tissue sections or in live cells.

Senescent cells can also be detected based on the signal pathways leading to their irreversible exit from cell cycle. In this regard, several markers are used, including increased phosphorylation of the Ser-139 residue of the histone variant H2AX (γ-H2AX), accumulation of tumor suppressor p53 and its downstream cyclin-dependent kinase (CDK) inhibitors (CKI) such as p21^Cip1^ (p21) and p16^Ink4a^ (p16), and increase of the senescence-associated heterochromatin foci, which manifests altered DNA packing within senescent cells. Senescent cells are well known to produce a host of proinflammatory and profibrotic cytokines and factors termed as the senescence-associated secretory phenotype (SASP). However, it remains to be determined what percentage of the cells positive for these markers are true senescent cells that never enter cell cycle again.

Aside from these positive markers, senescent cells cease to express proliferation-related markers such as Ki-67, a nuclear protein associated with cell proliferation. Another feature of senescent cells is the loss of the structural nuclear lamina protein lamin B1 (LMNB1). Of note, decrease of lamin B1 is also observed in apoptotic cells but not in quiescent cells. However, lamin B1 is degraded by caspase in apoptotic cells, whereas its reduction in senescent cells results from a decreased mRNA stability.

Senescence most likely occurs in a dynamic, step-wise fashion. It has been proposed that senescence proceeds with two stages: early senescence and full senescence (Childs et al., 2015). The early senescence is characterized by permanent cell cycle arrest and SA-β-GAL positivity, whereas full senescence is irreversible cell cycle arrest, SA-β-GAL positivity and SASP production and secretion.

Given the difficulty in identifying senescent cells, a three-step multi-marker workflow has been proposed recently for detecting senescent cells (Gorgoulis et al., 2019). The first step involves assessing SA-β-Gal activity, while the second step is to co-stain for additional senescent markers, such as p16, p21, *γ*-H2AX and LMNB1. In the final step, one needs to examine senescence markers that are expected to change in the biological context studied. Together, combinations of these methods provide a robust and sufficient approach for detection and monitoring of senescent cells in different settings.

### Classification of Cellular Senescence

Cellular senescence can be triggered by a wide variety of stressors, and senescent cells may exhibit immense diversity with different cell origins and varying degree of genomic and epigenomic instability. Depending on the developmental stage, different timing and distinct function, cellular senescence can be divided into three categories: developmental senescence, acute senescence and chronic senescence. Developmentally programmed senescence and acute cellular senescence are usually beneficial, as these senescent cells are generated by defined triggers, regulated by transient signaling and controlled by eventual clearance. However, chronic cellular senescence triggered by aging and sustained injury leads to permanent cell cycle arrest, resists to apoptosis and is unable to proliferate, ultimately causing tissue fibrosis and organ dysfunction (Schmitt and Melk, 2012; van Deursen, 2014; Childs et al., 2015).

Developmental senescence is believed to play a critical role in the fine-tuning of embryogenesis. Senescent cells are transiently present in multiple embryonic structures including the mesonephros, the neural tube, the developing limb, and the endolymphatic sac of the inner ear, as detected by SA-β-Gal staining, p21 expression and SASP activation. In developing kidney, senescent cell accumulation precedes macrophages infiltration, suggesting its role in attracting immune cells to facilitate mesonephros regression via clearance of the senescent cells. Loss of developmental senescence in p21^−/−^ knockout mice leads to compensatory increase in apoptosis. Although p21^−/−^ mice remain viable, they exhibit detectable abnormalities in their kidney, limbs and vagina (Munoz-Espin et al., 2013). These studies suggest a critical role of developmental senescence, as a mechanism that complements apoptosis, in eliminating specific groups of cells during embryogenesis.

Acute cellular senescence is induced as an integral part of the healing and regenerative response after an initial insult. Studies show an early induction of senescence in endothelial cells and fibroblasts in response to cutaneous injury (Demaria et al., 2014). This acute senescence is transient and promotes wound healing by secreting platelet-derived growth factor (PDGF). Senescent cell ablation delays, but does not prevent, wound healing. Senescent fibroblast accumulation is observed after skin wound or post myocardial infarction, which functionally limits tissue fibrosis. Similarly, senescence of hepatic stellate cells also appears to attenuate liver fibrosis following injury. Therefore, acute senescence, if it is transient and under tight control of immune clearance, is beneficial in reducing fibrosis and promoting tissue regeneration and recovery.

Chronic senescence, however, leads to progressive accumulation of senescent cells when clearance is insufficient, contributing to kidney aging and CKD. Evidence is mounting that increased numbers of senescent cells are not merely a biomarker of aging or previous injury, they contribute to maladaptive repair and promote tissue fibrosis and organ dysfunction, and therefore represent a novel therapeutic target. There is a close association between senescent cell accumulation and CKD severity (Melk et al., 2005; Schafer et al., 2018). In the kidney, tubular epithelial cells are the major source of senescent cells in CKD, although other cells may also be involved, depends on the location and type of injury (Sis et al., 2007; Valentijn et al., 2017). Senescent cells after chronic injury are resistant to apoptosis, persistently reside within the injured kidney and secrete proinflammatory and profibrotic SASP components, thereby promoting progressive damage.

### Pathways Involved in Cellular Senescence

**FIGURE 2:**
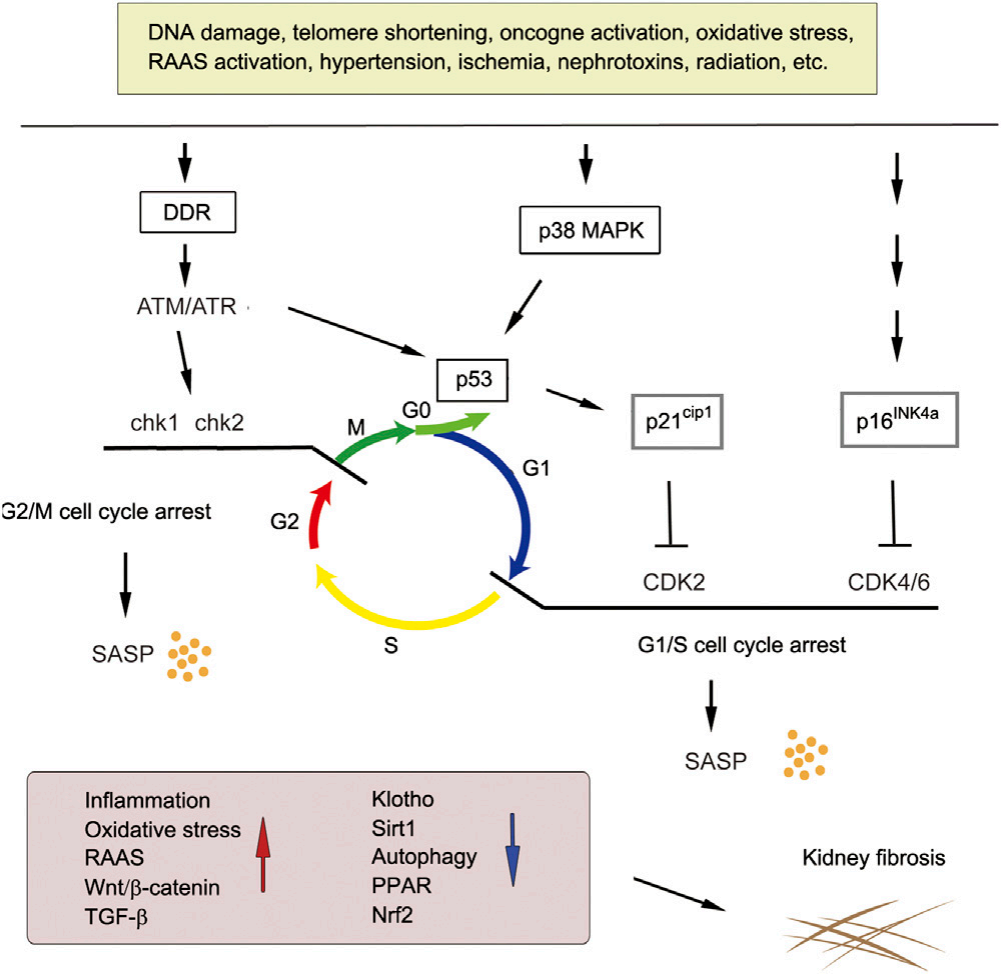
Major pathways leading to cellular senescence in kidney fibrosis. Various stressors induce cellular senescence via different signaling pathways. DNA damage, oncogene activation and oxidative stress trigger DNA damage response (DDR) and activates ataxia telangiectasia mutated (ATM) and ATM-Rad3-related protein (ATR) kinase, which induces G2/M cell cycle arrest via Chk1/2 activation or G1/S cell cycle arrest via p53/p21-mediated inhibition of CDK2 and/or CDK4/6. Senescent cells secret SASP to induce proinflammatory and profibrotic factors and repress Klotho, SIRT1, autophagy, PPAR and Nrf2 signaling, eventually leading to kidney fibrosis.

A diverse array of stressors can induce cellular senescence, such as telomere shortening, DNA damage, oncogenic stimuli, nephrotoxins, ischemia, mitochondrial dysfunction, ROS generation and mechanical stress. To ensure cell integrity after these stresses, DNA is checked during cell cycle progression in the G1/S or G2/M phases (Figure 2). Ataxia telangiectasia mutated (ATM) and ATM-Rad3-related (ATR) kinases play a central role in mediating DNA damage response (DDR). Both kinases rapidly phosphorylase the tumor suppressor p53 upon DNA damage, leading to the induction of p21^Cip1^. Furthermore, ATM also phosphorylates the checkpoint kinases Chk1 and Chk2. Phosphorylation of p21 inhibits CDK, leading to the dephosphorylation of retinoblastoma protein (Rb). This enables Rb to bind to transcription factor E2F, thereby leading to G1/S cell cycle arrest. Likewise, p38 MAPK activation induced by ROS and other stimuli also increases p53 activity. In addition to p53, accumulation of tumor suppressor p16^Ink4a^ also leads to cell cycle arrest via the inhibition of CDK4/CDK6 (Figure 2). Senescent cells produce and secrete SASP, leading to increased inflammation, oxidative stress, hyperactive RAAS, Wnt/β-catenin and TGF-β signaling, and decreased Klotho, Sirtuin 1 (SIRT1), peroxisome proliferator-activated receptors (PPARs) and nuclear factor erythroid 2-related factor 2 (Nrf2) signaling. These senescence-associated signaling such as RAAS, Wnt and TGF-β further promote secondary cellular senescence, creating a vicious cycle.

Senescence-provoking signaling can be different and involve distinct pathways between senescence programs. For instance, developmental senescence is mainly mediated by p21^cip1^, but does not involve a DDR and p53. Developmental senescence is also regulated by TGF-β/Smad and phosphatidylinositol 3-kinases (PI3K)/forkhead box protein O (FOXO) pathways, which can also be independent of p53 (Munoz-Espin et al., 2013). However, acute and chronic senescence triggered by various stressors often involve induction of p16^INK4A^ and activation of DDR and its downstream key effectors p53 and p21, which converge to inhibit CDKs (Figure 2). These studies suggest that developmental senescence is differentially regulated and is unlikely to be the consequence of cumulative cellular stress.

The p53 protein is strategically positioned and plays a vital role in cell fate determination after injury by an array of stressors. In the setting of DNA damage, the level of p53 expression may determine whether a cell cycle-arrested cell continues to replicate after the damage is repaired, or undergo cellular senescence or apoptosis. When DNA is irreparable, the fate of the cells is either apoptosis or senescence by permanently inhibiting cell cycle progression. Although the exact mechanisms under cell fate decision are still unclear (Valentijn et al., 2017), it is believed that the level of intracellular p53 may be the key. High level of p53 leads to apoptosis while lower level of p53 induces cellular senescence. In this regard, chronic senescence may represent a pro-survival choice of the cells after a sublethal injury.

### SASP

Senescent cells drive kidney fibrosis mainly by producing and releasing SASP, a collection of proinflammatory and profibrotic factors and cues. SASP forms a detrimental microenvironment that regulates the behavior of neighboring cells. SASP components include interleukin-1β (IL-1β), IL-6, IL-8, C-X-C motif chemokine ligand 1 (CXCL1), transforming growth factor-β1 (TGF-β1). They act in a paracrine and autocrine fashion and induce a series of downstream effects (Docherty et al., 2019). A study shows that treatment of human bronchial epithelial cells with serum from patients of chronic obstructive pulmonary disease induces senescent phenotype, including increased SA-β-Gal, γ-H2AX, and p21 (Kuznar-Kaminska et al., 2018). These results suggest that the extracellular factors produced by senescent cells may mediate non-senescent cells to cell cycle arrest, leading to secondary senescence. Of note, SASP produced by senescent cells exhibits some unique feature, which distinguishes it from that secreted by quiescent and other types of non-proliferating cells (van Deursen, 2014). As shown in Table 2, SASP components can be roughly divided into three major categories: soluble signaling factors such as interleukins, chemokines and growth factors, secreted proteases, and secreted insoluble proteins or extracellular matrix (ECM) ingredients (Coppe et al., 2010; Liu et al., 2020b).

**TABLE 2:** SASP composition and classification.

Subclasses	Factors
Interleukins (IL)	IL-6, IL-7, IL-1a, IL-1b, IL-13, IL-15
Chemokines (CXCL, CCL)	IL-8, GRO-a,-b,-g, MCP-2, MCP-4, MIP-1a, MIP-3a, HCC-4, eotaxin-3, TECK, ENA-78, I-309, I-TAC
Chemokines (other inflammatory factors)	GM-CSE, G-CSE, IFN-γ, BLC, MIF
Growth factors and regulators	Amphiregulin, epiregulin, heregulin, EGF, bFGF, HGF, KGF (FGF7), VEGF, angiogenin, SCF, SDF-1, PIGF, NGF, IGFBP-2, -3, -4, -6, -7
Soluble or shed receptors or ligands	ICAM-1, -3, OPG, sTNFRI, TRAIL-R3, Fas, sTNFRII, uPAR, SGF130, EGF-R
Nonprotein soluble factors	PGE2, nitric oxide, reactive oxygen species
Proteases and regulators	MMP-1, -3, -10, -13, -14, TIMP-1, TIMP-2, PAI-1, -2, tPA, uPA, Cathepsin B
Insoluble or ECM proteins	Fibronectin, collagens, laminin

The regulation of SASP expression is controlled at multiple levels including transcription, mRNA stability, protein translation and secretion. In addition, SASP forms a stable positive loop via autocrine and paracrine mechanism, which leads to signal amplification (Herranz and Gil, 2018). The expression of the majority of SASP is regulated by nuclear factor *κ*-B (NF-*κ*B), CCAT/enhancer binding protein-*β* (C/EBP-*β*), p38 mitogen-activated protein kinase (MAPK) (Flanagan et al., 2018). DNA damage and oxidative stress activate NF-*κ*B signaling, leading to overexpression of downstream SASP (Taniguchi and Karin, 2018; Wei and Ji, 2018). In kidney proximal tubular cells, indoxyl sulfate induces cellular senescence through reactive oxygen species (ROS)/NF-κB/p53 pathway, and NF-κB also suppresses cellular proliferation and upregulates TGF-β1 and α-SMA to induce fibrosis (Shimizu et al., 2010; Shimizu et al., 2011). P38 MAPK is another regulator of cell senescence. By activating p38 MAPK pathway, SASP can be induced by a mechanism independent of DDR (Alimbetov et al., 2016; Wei and Ji, 2018). C/EBP-*β* is upregulated in oncogene-induced senescence and it stimulates SASP expression (Lopes-Paciencia et al., 2019).

Although SASP components are relatively conserved and ubiquitous, senescent cells induced by different stressors may have some unique features with heterogeneous SASP, thereby inciting distinct responses in regulating cell proliferation, migration, inflammation and oxidative stress. Another characteristic of SASP is related to its temporal secretion, and not all factors are induced in the same time (Coppe et al., 2010). It is also unclear whether all SASP components are secreted as soluble factors or packaged in the extracellular vesicles including exosomes for efficient intercellular communication (Liu et al., 2020a). Further characterization of SASP composition, dynamics of its secretion, mode of its transmission and functional classification warrants more investigation.

## CELLULAR SENESCENCE IN CKD: TRIGGERS AND MECHANISM

### Senescence as an Injury Response

**FIGURE 3:**
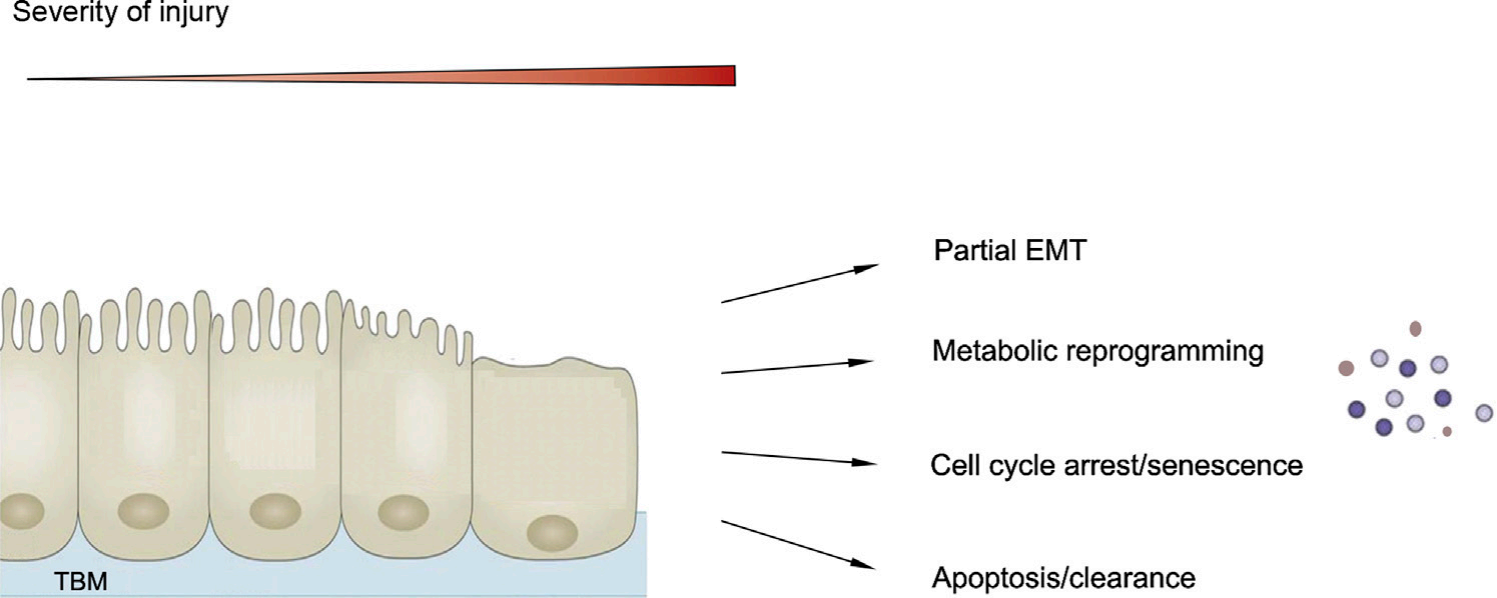
Senescence as one of the cellular responses after chronic kidney injury. Kidney cells, particularly proximal tubular cells, respond in different ways after chronic or repeated injury, ranging from partial epithelial-mesenchymal transition (EMT), metabolic reprogramming, cell cycle arrest and senescence to cell death. One common outcome of these changes including EMT, metabolic reprogramming and cell cycle arrest and senescence is converting these cells into secretory phenotype.

In response to oxidative, toxic and metabolic insults, kidney cells, particularly the proximal tubular epithelial cells, respond in different ways and undergo a spectrum of changes, such as partial epithelial-mesenchymal transition (pEMT), metabolic reprogramming, cell cycle arrest and cellular senescence, and various forms of cell death including apoptosis and necrosis (Figure 3) (Zhou and Liu, 2016). The majorities of these responses to sublethal injury are believed to be evolutionarily conserved programs in an effort of promoting survival and preventing cell death. However, if these responses are not under control or resolved promptly after chronic or repeated injury, they induce maladaptive changes and promote the initiation and progression of CKD. One shared and convergent consequence of these responses including pEMT, metabolic reprogramming and cellular senescence is converting the affected cells into secretory phenotype, thereby producing substantial amount of inflammatory and profibrotic factors and releasing into extracellular space to incite secondary responses (Figure 3).

As one of the injury responses, the exact relationship of cellular senescence to other responses after kidney injury remains elusive. Earlier studies suggest that pEMT precedes cell cycle arrest/senescence after kidney injury (Lovisa et al., 2015), suggesting a temporal relationship between these two events. At present, it is unclear how kidney cells decide to proceed through a special route in response to an insult. As aforementioned, the level of p53 may be an arbitrator in determining the cell fate such as apoptosis or senescence. More studies are needed in this area.

### Cellular Senescence in CKD

Senescent cells can be detected in different tissue compartments during aging and CKD. Generally, proximal tubular epithelium is the major site of senescent cells after kidney injury, although other sites such as glomeruli and endothelium also harbor senescent cells. It is commonly believed that different stressors that target varying cells may determine the localization and type of senescent cells. Table 3 lists the detection of senescent cells in various kidney diseases.

**TABLE 3:** The detection of senescent cells in various kidney diseases.

Renal defect	Organism/model	Senescence markers	Cell type affected	Potential effect	Refs	
**Aging kidney**						
	Human	p16^INK4a^, TP53, p27^KIP1^, p14^ARF^,	Cortical tubules, parietal epithelial cells and podocytes, interstitial cells and arteries	Detrimental	(Chkhotua et al., 2003; Melk et al., 2004; Melk et al., 2005)
	Mouse and rats	SA-β-Gal, p16^INK4a^, p19^ARF^, p21^CIP1^	Proximal tubules and glomerular podocytes	Detrimental	(Krishnamurthy et al., 2004; Baker et al., 2016; Miao et al., 2019)
**Kidney disease**						
AKI	Multiple mouse models	SA-β-Gal, p21^CIP1^, γ-H2AX, p16^INK4a^, p19^ARF^	Proximal tubular cells	Mostly detrimental	(Chkhotua et al., 2003; Yang et al., 2010; Clements et al., 2013)
	
IgAN	Human	SA-β-Gal, p21^CIP1^, p16^INK4a^	Renal tubular epithelial cells	Detrimental	(Liu et al., 2012)
DN	Human	SA-β-Gal, p16^INK4a^	Proximal tubular cells, podocytes, mesangial cells, arterial vessels	Detrimental	(Verzola et al., 2008)
	Mouse STZ	SA-β-Gal, p21^CIP1^	Mainly in cortex tubular epithelial cells	Detrimental	(Kitada et al., 2014)
MN, FSGS	Human	p21^CIP1^, p16^INK4a^	Parietal epithelial cells, podocytes, mesangial cells, tubular cell, interstitial cells, artery	Detrimental	(Sis et al., 2007)
Fibrosis	Mouse (IRI UUO, ADR model)	SA-β-Gal, p16^INK4a^, γ-H2AX	Renal tubular epithelial cells	Detrimental	(Luo et al., 2018)
**Therapy-induced**						
Renal transplantation	Human	SA-β-Gal, p16^INK4a^	Infiltrating cells in interstitial area, tubular cells, vascular cells	Detrimental	(Melk et al., 2004; McGlynn et al., 2009)

Aging kidneys have poor outcome when transplanted, and cellular senescence is a crucial prognosis for poorer graft survival (Melk, 2003; Sosa Pena et al., 2018). In a cohort of 75 cases with preimplantation renal allograft biopsies, the levels of cellular senescence marker p16^INK4a^ predicates postoperative renal function in humans (McGlynn et al., 2009). A retrospective clinical study also indicates that cellular senescence in kidney tubular epithelia of IgA nephropathy (IgAN) patients corelates with its progression (Liu et al., 2012). P16^INK4a^ positive cells can be found in the glomeruli and tubulointerstitium of renal biopsies from patients with minimal change disease (MCD), membranous nephropathy (MN), focal and segmental glomerulosclerosis (FSGS) (Sturmlechner et al., 2017). In type 2 DN, senescent cells are found in the glomerulus and renal tubules, which positively correlates with the severity of DN (Verzola et al., 2008).

Cell cycle arrest/senescence plays a critical role in mediating AKI-CKD progression (Clements et al., 2013). Using multiple animal models, investigators show that G2/M arrest in proximal tubular cells produces profibrotic factors through JNK pathway (Yang et al., 2010). Genetic ablation of p16^INK4a^ improves kidney regeneration and reduces capillary rarefaction after ischemic injury in p16^INK4a^ null mice (Lee et al., 2012). Furthermore, using transgene INK-ATTAC technology to selectively remove p16^INK4a^-positive senescent cells upon drug treatment attenuates age-related phenotypes and extends healthy lifespan (Baker et al., 2011; Baker et al., 2016). Epithelial cell-specific deletion of Myd88, an adapter protein in NF-κB signaling, reduces expression of proinflammatory cytokines such as IL-1α, IL-1β, IL-6, TNF-α, and MCP-1 after folic acid injury and attenuates renal fibrosis. This suggests that innate immune signaling also participates in cellular senescence after kidney injury (Jin et al., 2019).

### CKD-Associated Senescence Triggers

#### Oxidative Stress

There are plentiful stressors and cues that can trigger or promote cellular senescence in CKD. The most studied cue causing cellular senescence is oxidative stress, which results from an imbalance between free radicals and antioxidants in cells and tissues. Oxidative stress is prevalent in both aging kidney and CKD (O'Sullivan et al., 2017; Zhou et al., 2019b). Advanced glycation end products (AGEs) and advanced oxidation protein products (AOPPs) arise from not only chronic disease conditions such as hyperglycemia and CKD but also during aging (Vlassara et al., 2009; Frimat et al., 2017). AGEs and AOPPs mainly bind to and interact with RAGE, the receptor of AGEs, which then activates downstream pathways such as TGF-*β*, NF-*κ*B, mitogen-activated protein kinase (MAPK) and NAPDH oxidase (NOX) (Zhou et al., 2012; Ott et al., 2014; Chen et al., 2019). All these mediators contribute to the induction of multiple cytokines and growth factors, eventually causing premature aging and tissue damage.

Several signal pathways such as NOX, TGF-*β*, RAAS and Nrf2 are participated in oxidative stress, thereby implicating in kidney damage and fibrosis (Okamura and Pennathur, 2015; Chen et al., 2019). Among them, NOX family is particularly relevant as they are an important generator for ROS, which damages lipids, DNA and proteins and causes fibrosis in multiple organs, especially the kidneys (Yang et al., 2018). ROS also activates p53/p21 signaling, resulting in enhanced apoptosis and cellular senescence. Furthermore, studies indicate that ROS can cause telomere-associated genomic instability and senescence in mesenchymal stem cells (Estrada et al., 2013).

#### Inflammation

Aging and age-related diseases converge on inflammation. Inflammaging is a special term describing a chronic, sterile and low-grade inflammation that develops with advanced age. Systemic inflammation increases with age (Goldberg and Dixit, 2015) and is worsened in chronic diseases, such as metabolic syndrome (Hotamisligil, 2017), CKD (Kooman et al., 2017a) and cardiovascular disease (CVD) (Gistera and Hansson, 2017). Two main mechanisms involved in inflammaging are mitochondrial dysfunction and autophagy dysregulation, the former leads to release ROS and the latter produces more intracellular inflammatory irritants (Bennett et al., 2018). To some extent, autophagy in CKD is considered protective, as it can inhibit inflammation by the removal of dysfunctional mitochondria and inhibiting ROS generation (Kimura et al., 2017). There are similarities in chronic inflammation between CKD and aging, including oxidative stress and cellular senescence. Several inflammatory cytokines such as IL-6 and IL-8 are important mediators of cellular senescence and modulating these cytokines or their receptors regulates the generation and accumulation of senescent cells. In addition, an intense inflammatory response after kidney injury has been shown to control the size of senescent cell population (Ferenbach and Bonventre, 2015; Zhou et al., 2020).

#### Wnt/β-Catenin

Developmental signaling pathways such as Wnt, hedgehog and Notch play vital roles in kidney fibrosis (Zhou et al., 2014; Edeling et al., 2016; Zhou et al., 2019a). Among them, Wnt/β-catenin signaling is mostly studied and implicated in age-related kidney disease (Liu et al., 2007; Luo et al., 2018). Wnt/β-catenin is an evolutionarily conserved pathway that is crucial for cell fate determination, organ development and injury repair (Angers and Moon, 2009; Tan et al., 2014; Nusse and Clevers, 2017). Sustained activation of Wnt/β-catenin is critical in kidney fibrogenesis after acute kidney injury (AKI) (Xiao et al., 2016). Recent studies show that pro(renin) receptor (PRR) is an indispensable component of Wnt receptor complex and acts as an amplifier of Wnt/β-catenin (Li et al., 2017).

The connection between Wnt/β-catenin and aging is uncovered in Klotho-deficient mice, in which augmented Wnt signaling is linked to stem cell depletion (Liu et al., 2007). Wnt/β-catenin is activated in muscle of aged animals, which impairs muscle regeneration and induces muscle fibrosis (Brack et al., 2007). Wnt signaling is also activated in the hearts of aged animals (Zhao et al., 2019). However, whether age-related cardiac changes cause activation of Wnt signaling or inhibition of Wnt signaling could mitigate cardiac lesions needs further investigation (Naito et al., 2010). After kidney injury, Wnt9a is upregulated and induces tubular cell senescence. This effect can be amplified through a TGF-β/Wnt/β-catenin bidirectional stimulation (Luo et al., 2018). In aging kidney, activation of Wnt/β-catenin leads to RAAS activation (Zhou et al., 2015a), which in turn triggers mitochondrial dysfunction, promotes cellular senescence and exacerbates age-related kidney fibrosis (Miao et al., 2019).

#### RAAS

Another common regulator of aging and CKD is the hyperactive RAAS (Dinh et al., 2017). Angiotensin II (Ang II) induces renal inflammation and fibrosis through TGF-β activation. Besides TGF-β, connective tissue growth factor (CTGF), endothelin 1 (ET-1) and plasminogen activator inhibitor 1 (PAI-1), as well as inflammatory cytokines such as IL-6, vascular cell adhesion molecule 1 (VCAM-1), and tumor necrosis factor-α (TNF-α), are also involved in Ang II-induced kidney fibrosis (Ruster and Wolf, 2011; Forrester et al., 2018). Ang II/TGF-β pathway contributes ROS generation through NOX4 (Chen et al., 2019). Of interest, oxidative stress also induces renal RAAS overactivation, creating a vicious cycle. Aldosterone, another vital component of RAAS, directly induces renal proximal tubular EMT via generating mitochondrial ROS, which in turn decreases peroxisome proliferator-activated receptor-*γ* coactivator-1*α* (PGC-1*α*) and activate extracellular signal-regulated protein kinase-1/2 (ERK1/2) and Snail1 (Yuan et al., 2012; Balakumar et al., 2019). Blockade of RAAS activation is the mainstay therapy for CKD patients (Romero et al., 2015). In animal models of CKD and humans, angiotensin converting enzyme inhibitors (ACEIs), angiotensin receptor blockers (ARBs) and aldosterone blockers improve kidney function and ameliorate renal fibrosis (Romero et al., 2015).

#### mTOR

The mammalian target of rapamycin (mTOR) signaling is another pathway contributing to cellular senescence by stimulating SASP expression and activating NF-κB signaling. An *in vitro* study demonstrates that inhibition of mTOR reduces SASP mRNA transcription and translation (Herranz et al., 2015). The level of mTOR expression increases with aging in rat kidneys and mesangial cells, and its activation promotes cellular senescence by inducing the G1/S cell cycle arrest through p21 (Zhuo et al., 2009). In mammals, inhibition of mTOR prolongs lifespan and delays the onset of age-related diseases (Weichhart, 2018), suggesting the possibility of preventing human aging and age-related diseases with mTOR inhibitors.

## INTRINSIC INHIBITORS OF CELLULAR SENESCENCE

### Klotho

Given the importance of cellular senescence in determining organismal longevity, it is not surprising that this program is delicately controlled by both positive and negative regulators. There are several naturally occurring, endogenous anti-aging factors that negatively regulate cellular senescence and aging. Among them, Klotho is the most well-studied anti-aging protein. It is a transmembrane glycosidase with extracellular KL1and KL2 domains. Klotho is mainly expressed in kidney, brain and parathyroid glands (Ide et al., 2016). Although all tubular segments in the kidney express Klotho, its level in the distal convoluted tubules is the highest. Global or kidney-specific deletion of Klotho in mice exhibits a premature aging phenotype with short lifespan, growth retardation, decreased physical activity, ectopic calcification, atrophy of skin and internal organs (Kuro-o et al., 1997; Lindberg et al., 2014). In contrast, transgenic mice with overexpression of Klotho resist to pathogenesis and have longer lifespan than wild type counterparts (Kurosu et al., 2005).

The full-length, membranous form of Klotho (mKlotho) acts as a co-receptor for the fibroblast growth factor 23 (FGF23). Klotho and FGF receptor (FGFR) work together to mediate FGF23 action and play a crucial role in maintaining phosphate homeostasis. Besides mKlotho, there is a secreted, soluble form of Klotho (sKlotho), which is generated from either alternative mRNA splicing or proteolytic shedding of the extracellular domain of mKlotho. The sKlotho can be excreted into blood circulation, body fluid and urine, and therefore can act on distant organs as a hormone.

Loss of Klotho is a common finding after kidney injury, which plays a pathogenic role in the onset and progression of kidney disorders. In virtually all kidney disease models including UUO, DN, adriamycin nephropathy, remnant kidney after 5/6 nephrectomy (5/6NX), ischemia/reperfusion injury (IRI) and cisplatin nephropathy, the expression of Klotho is markedly decreased in serum and the kidneys (Zhou et al., 2013; Zhou et al., 2015b). In aging and age-related diseases such as CKD, diabetes, atherosclerosis, Alzheimer’s disease and cancer, Klotho is significantly lower than normal and healthy subjects (Koh et al., 2001; Bian et al., 2015). A clinical study on health aging and body composition (Health ABC) also reveals that higher sKlotho level in serum independently associates with a lower risk of decline in kidney function (Drew et al., 2017).

Klotho is known to promote potassium secretion into urine by upregulating the renal outer medullary potassium channel 1 (ROMK1) on the plasma membrane. It also regulates the transient receptor potential cation channel, subfamily V, member 5 (TRPV5), a calcium channel in renal tubular cells, causing Ca^2+^ reabsorption. Klotho inhibits insulin/insulin-like growth factor 1 (IGF-1) activity, which is related to its ability to decrease oxidative stress and prolong lifespan.

Mounting evidence suggests a special role of Klotho in preventing kidney fibrosis by targeting multiple key profibrotic signaling. Earlier studies show that Klotho directly binds to the type II TGF-*β* receptor (T*β*R2), which prevents its engagement with TGF-*β*1, thereby blocking TGF-*β*1 signaling (Doi et al., 2011). Studies from our laboratory show that Klotho intercepts Wnt/*β*-catenin signaling by binding to various Wnt ligands, thereby inhibiting Wnt signal transduction (Zhou et al., 2013). As Wnt/*β*-catenin plays a fundamental role in kidney fibrogenesis and cellular senescence (Zuo and Liu, 2018), inhibition of its signaling by Klotho results in alleviation of fibrotic lesions in various models of CKD (Zhou et al., 2013; Zhou et al., 2015b). Furthermore, Klotho has been shown to suppress renal tubulo-interstitial fibrosis by constraining FGF-2 signaling (Guan et al., 2014). These findings establish that Klotho explicitly targets TGF-*β*, Wnt and FGF-2, the three important signal pathways implicated in kidney fibrosis.

As an intrinsic anti-aging protein, Klotho may have a unique role in halting cellular senescence. The connection between loss of Klotho and increased cellular senescence is validated in many studies. Klotho reduces endothelial cell senescence induced by uremia through blocking NF-κB signaling (Zhao et al., 2011; Buendia et al., 2015). As Wnt/*β*-catenin signaling promotes kidney tubular epithelial cell senescence (Luo et al., 2018), Klotho can play an anti-aging effect by downregulating Wnt signaling. Along this line, loss of Klotho in CKD de-represses Wnt signaling, leading to an exaggerated tubular cell senescence, kidney fibrosis and dysfunction. A recent study indicates that high phosphate induces kidney tubular epithelial cell senescence, which is blocked by Klotho (Maique et al., 2020). Furthermore, hyperphosphatemia induced by Klotho depletion in CKD is a trigger to renal osteopathy and vascular calcification, which may indirectly accelerate aging and increase mortality (Kuro-o, 2011).

### Sirtuin1

Sirtuins are an evolutionarily conserved family of NAD^+^-dependent deacetylases and regulate the stress response programs by modulating many crucial regulators, such as NF-κB, p53, hypoxia-inducible factor 1α (HIF-1α), FOXOs, PGC-1α (Hekmatimoghaddam et al., 2017; Ryu et al., 2019). There are seven sirtuins (SIRT1-7) localizing in different tissues and subcellular compartments. Through controlling key transcription factors via epigenetic regulation, they play a critical role in the development and progression of a wide variety of diseases, such as premature aging, CKD, metabolic syndrome and cancer (Huang et al., 2017).

SIRT1 is the prototypic member of the sirtuins family and one of the most well-characterized sirtuins. In the kidney, SIRT1 is mainly expressed in tubular epithelial cells and glomerular podocytes. SIRT1 is renal protective and prevents renal lesions after various injuries. The beneficial effects of SIRT1 are mediated by its ability to inhibit renal fibrosis, reduce tubular and glomerular cell apoptosis and inflammation, promote autophagy and control blood pressure (Kitada et al., 2013). In AKI and DN models, SIRT1 stabilizes mitochondrial function, activates PGC-1α/PPARγ and FOXO3/FOXO4 signaling in tubular cells, and reduces podocytes damages through acetylating of the p65 NF-κB and STAT3 (Yacoub et al., 2014). In CKD induced by UUO, SIRT1-deficient mice exhibit more severe renal fibrosis and apoptosis compared with wild-type controls (He et al., 2010). In a study with podocyte-specific SIRT1 knockdown mice, aged mice display more accumulation of senescent cells and severer sclerosis in glomerulus compared to controls, and this effect is mediated by an increased acetylation of FOXO3, FOXO4, NF-κB and PGC-1α (Chuang et al., 2017). Therefore, loss or dysregulation of SIRT1, an intrinsic inhibitor of aging, promotes cellular senescence and CKD progression.

## THERAPEUTIC INTERVENTION STRATEGY

### Senolytic

**FIGURE 4:**
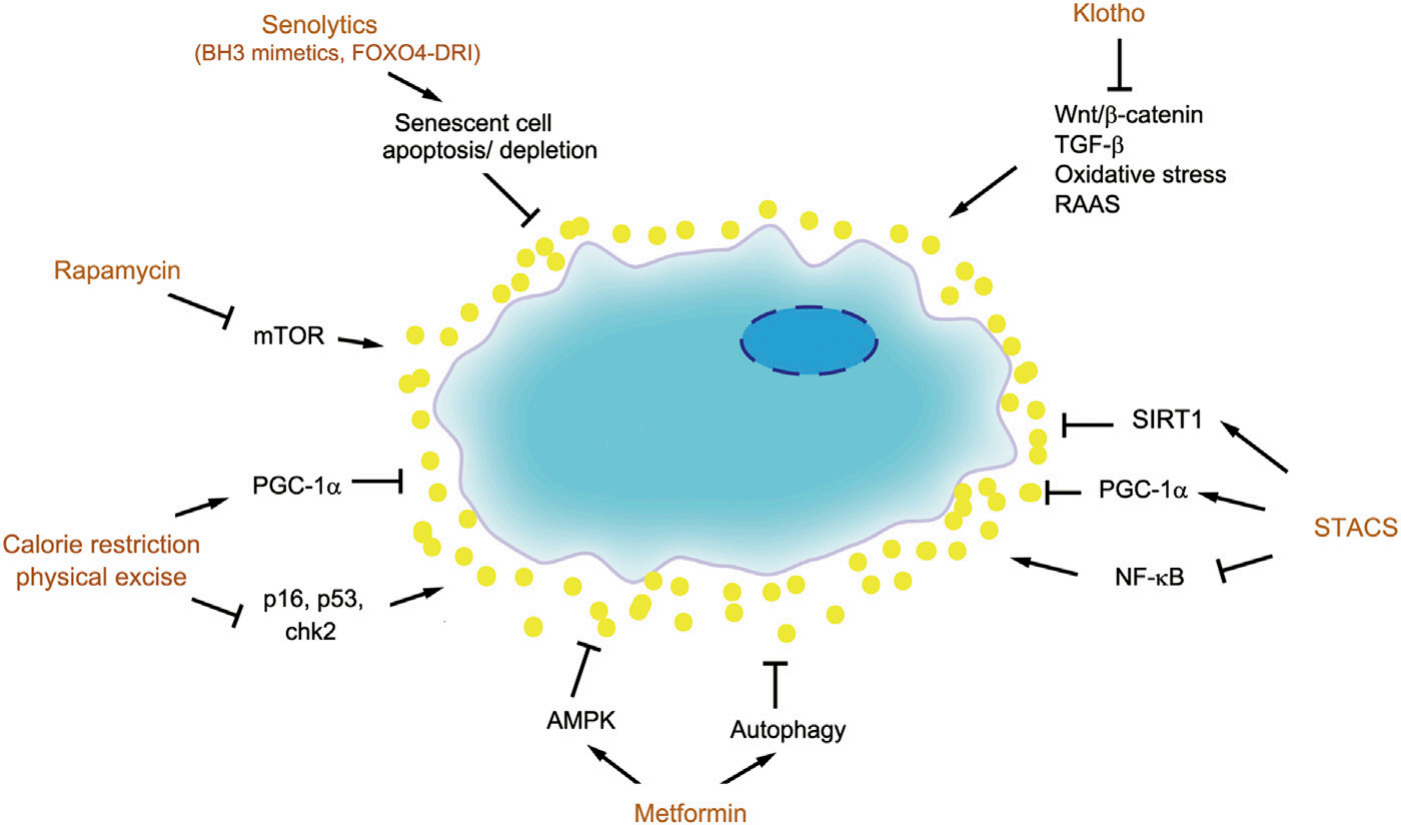
Therapeutic strategies for targeting senescent cells. There are several therapeutic strategies to target senescent cells, such as senolytics (BH3 mimetics and FOXO4-DRI), senostatics including rapamycin, metformin, calorie restriction and physical exercise, as well as boost of senescence inhibitors such as Klotho and SIRT1.

As senescence plays a crucial role in aging and disease, senescent cells have emerged as promising new target for therapeutic intervention known as senotherapy. The senotherapy includes depleting senescent cells (senolytic), suppressing SASP (senostatic) and boost/restoration of senescence inhibitors (Figure 4). The goal of senotherapy is to develop agents or strategies to prevent, alleviate, or reverse age-related diseases.

The senolytic approach to remove senescent cells for preventing aging and age-related disease is initially conceptualized based on the studies on transgenic mice. To deplete senescent cells selectively, the promoter of the p16^INK4a^ gene is used to drive the expression of a suicide gene which can be activated by administration of a drug or dietary compound. Using this approach, a landmark paper demonstrated successful elimination of senescent cells *in vivo* as shown by SA-β-Gal staining (Baker et al., 2016). The kidney of mice with senescent cell depletion show significant protection with less glomerulosclerosis and lower blood urea levels. A similar approach using the P16:3MR mouse model, where p16^INK4a^ promoter drives the expression of a viral thymidine kinase, conferring selective sensitivity to the effects of the anti-viral drug ganciclovir (Baar et al., 2017). Mice with senescent cell depletion had lower levels of serum creatinine and blood urea nitrogen. These studies provide a proof-of-principle for the feasibility of senescent cell depletion in preventing age-related disease (Baker et al., 2011).

To translate these studies on mouse genetic models to clinical setting, pharmacological approaches capable of targeting senescent cells *in vivo* are needed. Senescent cells typically resist to apoptosis and enter the permanent cell cycle arrest by upregulating pro-survival proteins such as Bcl-2, Bcl-xL and Bcl-w. ABT-263, also known as navitoclax, and related compounds ABT-737 and ABT-199 are Bcl-2/xL/w inhibitors, which act as BH3 mimetics and occupy the BH3-binding groove of these pro-survival proteins, thereby preventing their binding to pro-apoptotic proteins BAX and BAK (Knoppert et al., 2019). This enables BAX and BAK to release and form oligomers and initiate the caspase cascade leading to apoptosis. Studies show that BH3 mimetics leads senescent cell to apoptosis, promotes tissue repair and rejuvenates aged stem cells. This observation is confirmed in two mouse models including ionizing radiation-induced lung damage and senescence and a transgenic model of senescence in the skin (Yosef et al., 2016). Whether this approach is efficacious in CKD models remains to be tested. One concern is the specificity of this approach. Whether the BH3 mimetics merely work in the senescent cells, but not in other non-senescent cells, remains to be carefully validated.

The p53 is an arbitrator dictating cell apoptosis or senescence, and its activity is subjected to regulation by FOXO4 protein. By binding to p53, FOXO4 prevents senescent cells from apoptosis. FOXO4-DRI (FOXO4-D-Retro-Inverso) is an interfering peptide that competes with endogenous FOXO4 for p53 binding, thereby enabling senescent cells to undergo p53-mediated apoptosis (Baar et al., 2017). Treatment with FOXO4-DRI reduces senescent cells in the kidney as assessed by SA-β-Gal staining and loss of LMNB1 in a genetic model of fast-aging mice or CKD model induced by adriamycin. FOXO4-DRI also preserves kidney function and inhibits IL-6 expression (Baar et al., 2017). These studies indicate that an increase of p53 by disrupting its binding to FOXO4 results in selective clearance of senescent cells in diseased kidneys, leading to renal protection. It remains to be seen whether this senolytic approach also works in other age-related disease models.

### **Senostatic**

Senescent cells drive tissue fibrosis and CKD progression mainly by secreting proinflammatory and profibrotic SASP components. As such, a potential strategy for senotherapy is to modulate or impede senescent cells to produce and release deleterious SASP, instead of killing senescent cells themselves. This kind of “senostatic” approach may be less specific, in view of that the SASP is context-dependent and dynamic. However, one advantage is the fact that there are many drugs or strategies currently available to modulate SASP production and secretion. Therefore, repurposing these existing drugs as senostatics would greatly speed up their clinical translation.

#### Rapamycin

Rapamycin is a specific inhibitor of mTOR, which has been used in clinic as anti-rejection drug after organ transplantation. Of interest, rapamycin prolongs lifespan in multiple animal models (Kapahi et al., 2004; Johnson et al., 2013; Weichhart, 2018). Aged mice fed with rapamycin have an extended lifespan, high survival rate and less pathological lesions (Harrison et al., 2009; Zhang et al., 2014). In senescent cells, activation of mTOR promotes SASP secretion, while rapamycin blocks the proinflammatory phenotype. The activity of mTOR is notably increased in normal aging kidney and senescent mesangial cells and fibroblasts. Activation of mTOR is known to promote the expression of p21, thereby mediating cell cycle arrest and SASP secretion, which could be blocked by rapamycin (Zhuo et al., 2009).

Several studies have confirmed the efficacy of rapamycin in ameliorating kidney fibrosis in various animal models of CKD such as experimental membranous nephropathy (Bonegio et al., 2005), anti-thy1-induced progressive glomerulosclerosis (Kramer et al., 2008), UUO (Wu et al., 2006) and IRI models (Jain et al., 2001). Due to the inhibition of T cell proliferation, rapamycin and its analogues are widely used in clinic as an immunosuppressive in kidney transplantation and glomerulonephritis (Jolicoeur et al., 2003; Fantus et al., 2016). Although blocking mTOR by rapamycin prolongs lifespan and mitigates age-related diseases, side effects such as immunosuppression, hyperglycemia and dyslipidemia should be monitored.

#### Metformin

Metformin is an effective and widely used anti-hyperglycemia drug. Aside from treating diabetes, metformin has been found to have numerous beneficial effects in many tumors including breast, liver, ovarian/endometrial and pancreatic cancers (Morales and Morris, 2015). Metformin specifically blocks the mitochondrial respiratory chain complex 1, which disrupts the balance between adenosine triphosphate (ATP) production and consumption, leading to an increased ratio of adenosine monophosphate (AMP) to ATP and further activating 5’-AMP-activated protein kinase (AMPK). AMPK is an evolutionarily conserved protein kinase which regulates energy balance and protects cellular function. Activation of AMPK can ameliorate myocardial, hepatic, pulmonary, and renal fibrosis (Jiang et al., 2017). Except for AMPK, mTOR and insulin/IGF-1 signaling are also the targets of metformin (Bao et al., 2014).

In kidney diseases, metformin also shows protective and therapeutic effects. Studies show that metformin attenuates renal fibrosis in UUO model by inhibiting ERK signaling and reducing the production and deposition of ECM proteins. In renal fibroblasts, metformin blocks Ang II-induced ERK activation and ECM expression (Shen et al., 2016). In adenine-induced uremic rat, metformin is able to protect against kidney damage and CKD-related mineral and bone disorders and attenuates vascular calcification (Neven et al., 2018).

#### Nrf2 Activator

As oxidative stress is a potent trigger for cellular senescence, restoration of the balance between free radicals and antioxidants is a potential strategy for limitation of senescent cell accumulation. The Nrf2 is an emerging regulator of cellular resistance to oxidants, which is a transcription factor regulating many target genes including antioxidant factors (Stenvinkel et al., 2018). In multiple models of CKD, the activity of Nrf2 is depressed, and therefore activating Nrf2 may represent a plausible approach to treat CKD. As summarized in a review article, Nrf2 is regulated by Keap1, GSK-3β, Bach1 and p53 during aging and cellular senescence (Silva-Palacios et al., 2018). Oxidant-induced preconditioning (OIP) strategy activates the cytoprotective Nrf2 and suppresses post-ischemic p21 induction and renal senescence in IRI mice (Johnson and Zager, 2018). Bardoxolone methyl is known to be one of the most effective agonists of Nrf2, which binds to Keap1 to cause Nrf2 to translocate into the nuclei and upregulate the expression of antioxidant factors. Bardoxolone methyl is effective for renal protection in animal models of CKD and in some clinical trials such as BEAM and TSUBAKI studies (Yamawaki et al., 2018). However, the BEACON study was prematurely terminated because of the higher rates of heart failure events in Bardoxolone methyl group (de Zeeuw et al., 2013). Identification of new Nrf2 activator with less side effects or modification of treatment scheme is warranted in the future.

#### Calorie Restriction

Calorie restriction (CR) and physical exercise can reduce cellular senescence and prolong healthy lifespan (Cartee et al., 2016; Souza et al., 2018). A study on weight loss due to CR relieves cell senescence in white adipose tissue (List et al., 2016). Through a 20-year study in primates, investigators show that CR exhibits benefits for age-related disease and prolongs lifespan (Colman et al., 2009). The effects of CR are likely to be related to its induction of AMPK and SIRT1 signaling and autophagy, while CR reduces inflammation, oxidative stress and mTOR signaling (Wang et al., 2018). Diet therapy has been an indispensable strategy for CKD management. Whether CR and weight loss will benefit for CKD needs further well-designed clinical trials.

Aerobic exercise can improve CKD-induced muscle wasting by inhibiting the overexpression of inflammatory factors, reducing oxidative stress, stabilizing mitochondrial function and autophagy (Zhang et al., 2019). Physical exercise upregulates telomerase activity in aorta and circulating mononuclear cells, downregulates p16, p53 and chk2 in mouse model. In hypertensive rats, exercise training reduces renal fibrosis by downregulating inflammatory and profibrotic pathways (Huang et al., 2018).

### Boost of Intrinsic Senescence Inhibitors

#### Klotho

As aging and age-related disease is associated with loss of endogenous Klotho, one strategy is to supplement exogenous Klotho or restore endogenous Klotho expression. A recent study demonstrates that Klotho abolishes proximal tubular epithelial cell senescence induced by high phosphate (Maique et al., 2020). Stable delivery of adeno-associated virus (AAV) expressing sKlotho to Klotho deficient mice ameliorates hyperphosphatemia and relieves vascular calcification (Hum et al., 2017). In an immune complex-mediated glomerulonephritis (ICGN) mouse model, Klotho transgene improves animal survival, preserves renal function, ameliorates glomerular and tubulointerstitial lesions by inhibiting mitochondrial DNA damage, oxidative stress and cellular senescence (Haruna et al., 2007). Klotho transgene therapy also improves kidney function and reduces tubulointerstitial damages induced by long-term infusion of angiotensin II (Mitani et al., 2002). In ischemic AKI model, Klotho decreases at acute phase, and together with high phosphate challenge promotes AKI to CKD progression, while Klotho improves AKI recovery and attenuates renal fibrosis by maintaining normal autophagy (Shi et al., 2016). In CKD models induced by UUO or adriamycin, loss of Klotho expression leads to activation of Wnt/β-catenin and upregulation of profibrotic proteins. In contrast, supplementation with Klotho in these two models inhibits renal fibrosis by intercepting Wnt/β-catenin signaling (Zhou et al., 2013). These studies suggest that supplementation of exogenous Klotho is an effective therapeutic strategy for treatment of CKD and age-related diseases.

Beside supplementation with exogenous Klotho, another approach is to induce or restore endogenous Klotho expression. In aging and CKD, Klotho gene is subjected epigenetic regulation via promoter CpG hypermethylation, and specific inhibition of DNA methyltransferase by 5-aza-2'-deoxycytidine causes demethylation of the Klotho gene and increases Klotho expression (Sun et al., 2012). In addition, TGF-β1 inhibits Klotho expression via p53-mediated miR-34a, which downregulates Klotho through direct binding with the 3' UTR of Klotho (Liu et al., 2019). In this regard, strategies to block TGF-β signaling would result in induction or restoration of Klotho in diseased kidneys. It is reasonable to speculate that some therapeutics such as sulodexide and dihydromyricetin mitigate CKD by enhancing Klotho expression (Kan et al., 2019; Liu et al., 2019).

#### STACs

Another strategy to boost endogenous anti-aging factors is using sirtuins-activating compounds (STACs). Up to date, several natural and synthetic STACs have been tested and proven to be effective for renal protection in various models of kidney diseases such as UUO, IRI, sepsis-associated AKI, cisplatin nephropathy and hypertensive nephropathy. Compounds that boost NAD^+^, including nicotinamide riboside and nicotinamide mononucleotide, and exogenous NAD^+^ make up a new class of STACs with therapeutic potential (Guan et al., 2017; Morigi et al., 2018).

Resveratrol is a flavonoid, which has multiple functions in protecting against aging and age-related diseases, such as CKD, coronary and cerebrovascular diseases, diabetic mellites, cancers, hypertension, and non-alcoholic fatty liver disease. In CKD, the mechanisms of resveratrol action include antioxidant activity, activating SIRT1 and PGC-1α and protecting mitochondrial function, anti-inflammation through suppressing TNF-α and NF-κB and inhibition of mTOR (Saldanha et al., 2013; Diaz-Gerevini et al., 2016). In aging kidney, resveratrol suppresses Ang II/AT1R axis and actives Mas receptor axis to reduce oxidative stress, inflammation and renal fibrosis (Jang et al., 2018). In another study, resveratrol protects aging kidney by activating the Nrf2, SIRT1 and AMPK signaling (Kim et al., 2018). Although resveratrol plays broad roles in a variety of age-related diseases, large-scale, high quality randomized controlled trials (RCTs) are needed to validate its effectiveness.

## Conclusion

Over the last several years, there is increasing appreciation that aging kidney and CKD share many common features, from clinical manifestation, pathologic presentation to underlying mechanisms. Accumulation of senescent cells and accompanied SASP have been identified as the major driver of renal fibrosis in age-related or diseased-associated kidneys. The importance of senescence in the pathogenesis of CKD has been elegantly illustrated in several genetic mouse models in which selective depletion of senescent cells ameliorates kidney pathology and prolongs healthy lifespan.

There are many new questions and knowledge gaps that need to be addressed. Renal proximal tubular epithelial cells, as the epicenter of injury, are frequently affected in aging and disease. Detailed characterization of these cells such as SASP composition and dynamics of secretion after various injuries in CKD patients are needed. Furthermore, as cellular senescence has been reported in all renal cells including endothelial cells and fibroblasts, the properties of senescence in these non-tubular cells deserves further investigation. Perhaps more importantly, how to translate our current knowledge into clinical practice is a daunting task. We are cautiously optimistic that different schemes of the senotherapies will eventually translate into new and efficacious strategies in fighting against CKD and other age-related diseases.

## Author Contributions

JX planned the study, reviewed the literature, prepared the figures and wrote the manuscript. LZ reviewed and revised the text. YL planned the study and revised the figures and text.

## Funding

This research was funded by the National Natural Science Foundation of China grant 81521003 and 81920108007.

## Conflict of Interest

The authors declare that the research was conducted in the absence of any commercial or financial relationships that could be construed as a potential conflict of interest.
